# A health economic analysis of an integrated diabetes care program in China: based on real-world evidence

**DOI:** 10.3389/fpubh.2023.1211671

**Published:** 2023-12-19

**Authors:** Di Liang, Wenjun Zhu, Jiayan Huang, Yin Dong

**Affiliations:** ^1^School of Public Health, Fudan University, Shanghai, China; ^2^Key Lab of Health Technology Assessment, National Health Commission, Shanghai, China; ^3^The People’s Hospital of Yuhuan, Taizhou, China

**Keywords:** diabetes, integrated care, primary care setting, health economic analysis, real-world evidence

## Abstract

**Introduction:**

An integrated care program was set up in China to improve the collaboration between primary healthcare centers and hospitals on diabetes management. This study aims to evaluate the economic value of this program with real-world data and to examine whether it can be promoted in primary healthcare settings in China.

**Methods:**

This integrated diabetes care program was implemented in Yuhuan City, China, to coordinate primary care and specialty care, treatment and prevention services, as well as the responsibilities of doctors and nurses. Cost-effectiveness analysis was used to compare the short-term economic value of this program (intervention group) versus usual diabetes management (control group). The cost data were collected from a societal perspective, while the effectiveness indicators pointed to the improvement of control rates of fasting blood glucose (FBG), systolic blood pressure (SBP), and diastolic blood pressure (DBP) levels after the 1 year intervention. In addition, cost-utility analysis was applied to evaluate the long-term value of the two groups. Patients’ long-term diabetes management costs and quality-adjusted life years (QALYs) were simulated by the United Kingdom Prospective Diabetes Study Outcomes Model 2.

**Results:**

The results showed that for 1% FBG, SPB, and DBP control rate improvement, the costs for the intervention group were 290.53, 124.39, and 249.15 Chinese Yuan (CNY), respectively, while the corresponding costs for the control group were 655.19, 610.43, and 1460.25 CNY. Thus, the intervention group’s cost-effectiveness ratios were lower than those of the control group. In addition, compared to the control group, the intervention group’s incremental costs per QALY improvement were 102.67 thousand CNY, which means that the intervention was cost-effective according to the World Health Organization’s standards.

**Discussion:**

In conclusion, this study suggested that this integrated diabetes care program created short-term and long-term economic values through patient self-management support, primary care strengthening, and care coordination. As this program followed the principles of integrated care reform, it can be promoted in China. Also, its elements can provide valuable experience for other researchers to build customized integrated care models.

## Introduction

Diabetes can have significant economic impacts on individuals, families, and society (1). It was estimated that 536.6 million adults had diabetes worldwide in 2021 (2). The direct and indirect costs of diabetes in 2015 were calculated to be US$1.31 trillion globally, accounting for 1.8% of the global Gross Domestic Product (GDP) in the same year (3).

Like the rest of the world, China also faces a significant burden of disease from diabetes. However, there are several challenges in managing diabetes in China. First, the capacity of primary healthcare centers (PHCs) is insufficient. According to a national survey, only half of the patients with diabetes can be diagnosed, and less than 10% of diagnosed patients had good diabetes control when they sought care from PHCs (4). Second is that secondary and tertiary hospitals undertake lots of routine diabetes management tasks instead of PHCs, which is a huge workload for hospitals’ medical staff. In summary, the collaboration between PHCs and hospitals is still underdeveloped in China.

In order to address these problems, an integrated diabetes care program called *Metabolic Management Center (MMC)* was set up. This program established a standardized clinical pathway that specified the responsibility of diabetes management at each level of medical facilities, and integrated the treatment and prevention services provided by PHCs and hospitals. In addition, through comprehensive training, the program also enabled medical staff to provide high-quality and continuous services to patients. The efficacy of the MMC program has been validated by previous randomized controlled trials (5). However, there is a relative paucity of studies investigating the effectiveness and economic value of this integrated diabetes care program based on real-world data.

Globally, debate continues about the economic value of integrated diabetes care programs. Some studies found that integrated care programs were cost-saving considering patients’ long-term coronary heart disease risk reduction (6). However, most studies suggested that integrated care programs can improve patients’ quality-adjusted life years (QALYs) but with additional input. The types and frequencies of disease management inventions included in integrated care programs would influence whether those programs were cost-effective or not (7). Furthermore, there were also some weaknesses in previous economic evaluations. First, the costs and clinical parameters required by the health economic analysis were normally collected from registered datasets, clinical trials, key informant interviews, and literature (8–11). Few studies utilized claim datasets to capture the real-world costs of diabetes treatment (12). Second, the perspectives of cost collection were mainly from the health system or third-party payer. Few studies collected patients’ direct non-medical costs and indirect costs. However, indirect costs were estimated to account for 34.7% of the global economic burden of diabetes in 2015 (3), so these costs were also essential and can largely influence patients’ lives.

In order to supplement the evidence for the economic value of diabetes care, our study conducted a health economic analysis of the aforementioned integrated diabetes care program in China. Real-world data was used to assess the short-term and long-term economic value of this program in order to evaluate whether it can be promoted in primary healthcare settings.

## Methods

### Study setting

Our study was conducted in Yuhuan City, Zhejiang Province, China. Yuhuan is located in southeast China, with a population of 648 thousand residents (12). In 2021, the yearly GDP *per capita* of Yuhuan was 110,122 Chinese Yuan (CNY) which is the currency of China (13), which was 35.99% higher than the GDP *per capita* in China (80,976 CNY) (14). Yuhuan has two secondary hospitals and 11 primary healthcare centers (PHCs). PHCs are responsible for the preventive care and treatment of common diseases, such as diabetes and hypertension. In contrast, the responsibilities of secondary hospitals mainly include treatment for specialty diseases. In order to promote integrated care, these medical facilities have integrated into two medical groups according to the geographic locations. Group 1 consists of one secondary hospital and five PHCs, and provides services for residents living in the southern part of Yuhuan. Group 2 contains the remaining facilities and serves residents living in the northern part of Yuhuan. However, since China does not establish formal gate-keeping mechanisms, patients can choose any medical facility to treat diseases based on their preference.

Group 1 initiated an integrated diabetes care program and was selected as the survey site in this study.

### Usual diabetes management

As part of the Essential Public Health Services in China, primary care providers (PCPs) in PHCs are responsible for diabetes management for patients with diabetes in Yuhuan City. Usual diabetes management consists of blood glucose surveillance, treatment plan adjustment, and health education. The interval of follow-up depends on patients’ disease risks, which are assessed by patients’ blood glucose levels and the presence of comorbidities or complications. For high-risk patients, the interval is 1 month, while for low-risk patients the interval is 3 months. When PCPs cannot handle patients’ problems, they would refer those patients to secondary hospitals. In practice, due to the limited capacity of PCPs, they can hardly address patients’ needs if complications arise. Consequently, many patients bypass PCPs and go directly to secondary hospitals due to the lack of gate-keeping mechanisms. However, there is still insufficient collaboration and coordination between PHCs and hospitals.

### Intervention

To supplement the shortcomings of usual diabetes management, an integrated diabetes care program (MMC program) was established. This program was delivered by two MMC centers in the secondary hospital and one of five PHCs in Group 1. This program aims to provide better diabetes management services for patients. Compared to usual diabetes management, MMC first integrated diabetes treatment and prevention services. MMC established a standardized management plan, which specifies detailed examination requirements (e.g., blood glucose, blood lipid, urine microalbumin) at each follow-up. This plan emphasizes the importance of both diabetes control and the prevention of diabetes-related complications, with the aim of early detection and treatment for retinopathy, nephropathy, neuropathy, vascular disease, foot ulcer, and so on. In addition, each MMC center has a health education nurse to provide personalized lifestyle interventions to patients. An App was also developed to encourage patients to record their self-tested fasting blood glucose (FBG) and blood pressure levels which can be supervised by physicians.

Second, MMC integrates primary care provided by the PHC and specialty care provided by the secondary hospital. The responsibilities of patient management are separated between the two facilities. Most follow-ups are done by PHCs, while patients only need to go to the hospital once a year. Furthermore, a referral network was built and all medical records of registered patients can be checked by responsible physicians in the secondary hospital and PHC. The divisions between nurses and doctors were also adjusted in two facilities, as nurses undertake many management responsibilities and doctors’ work burden is reduced.

### Study design

A cohort study was conducted to evaluate the economic value of the MMC program versus usual diabetes management. The study period ranged from May 1st, 2021 to April 30th, 2022. All patients with diabetes or pre-diabetes who registered at MMC from January to April 2021 in Yuhuan were included in the intervention group. The registration for MMC is based on patients’ willingness. On the other side, all patients with diabetes or pre-diabetes who received the usual diabetes management provided by five selected PHCs and did not register at MMC by the end of the study period were included in the control group. Both groups had the freedom to visit any PHCs or hospitals as needed.

The exclusion criteria for the study were: (1) patients who did not attend any social medical insurance in Yuhuan, which includes Basic Medical Insurance for Urban Employees (UE) and Basic Medical Insurance for Urban and Rural Residents (URR); (2) patients who did not have any diabetes-related records in claims dataset; (3) patients with no follow-up data after the one-year intervention period. According to these criteria, 295 patients from the intervention group and 6,435 patients from the control group were eligible.

This study was approved by the Medical Research Ethics Committee, School of Public Health, Fudan University (IRB#2021-TYSQ-04-122).

### Cost-effectiveness analysis

#### Cost parameters

The costs of intervention and control groups were collected from a societal perspective, which included direct medical costs, direct nonmedical costs, indirect costs, and overhead costs.

Direct medical costs pointed to the medical fees generated for the treatment of diabetes and its complications in any medical facilities around China, including reimbursed costs and patients’ out-of-pocket costs. These costs were retrieved from Yuhuan’s social health insurance claims dataset.

Direct nonmedical costs and indirect costs referred to transportation fees, hotel fees, and productivity loss due to diabetes treatment, which were gathered through patient questionnaires. This questionnaire survey, which was conducted by trained investigators, collected patients’ and their companions’ transportation fees, hotel fees, and time used to seek outpatient and inpatient care because of diabetes. The calculation of indirect costs (productivity loss) was based on the treatment time and the patient’s average daily income in Yuhuan. All participants were informed and consented to attend the survey.

Lastly, the study calculated the overhead costs for two groups, which mainly included the costs for health education, fee-for-free follow-ups, medical staff training, and clinic re-decoration. Those costs were estimated through interviews with six key informants who participated in usual diabetes management or MMC, including two hospital managers, two doctors, and two nurses. The summary costs were shared by each beneficiary patient.

In summary, the total costs for a patient’s 1 year diabetes management are equal to:


$$\begin{array}{l} Totalcosts=directmedicalcosts+directnonmedicalcosts\\ \;\;\;\;\;\;\;\;\;\;\;\;\;\;\;\;\;\;\;\;\;\;\;\;\;+indirectcosts+sharedoverheadcosts \end{array}$$

#### Short-term clinical parameters

The clinical effectiveness of intervention and control groups was evaluated in one year from May 1st, 2021 to April 30th, 2022. And the intervention and control groups’ data came from the MMC information system and county patients’ electronic health records, respectively. We measured the rate of patients who achieved FBG, systolic blood pressure (SBP), and diastolic blood pressure (DBP) control targets (control rate) at baseline and after intervention in two groups. The control targets for FBG, SPB, and DBP were <7.0 mmol/L, ≤139 mmHg, and ≤89 mmHg, respectively (15–17). The effectiveness indicators included the percentage changes in FBG, SPB, and DBP control rates, defined as:


$$\mathrm{Control rate}=\frac{\mathrm{No}.\mathrm{of patients}\;\mathrm{who}\;\mathrm{achieved control target}}{\mathrm{total number of patients}}$$

$$\begin{array}{l} Percentageofimprovement=controlrateafterintervention\\ \;\;\;\;\;\;\;\;\;\;\;\;\;\;\;\;\;\;\;\;\;\;\;\;\;\;\;\;\;\;\;\;\;\;\;\;\;\;\;\;\;\;\;\;\;\;\;\;\;\;\;\;\;\;\;\;\;\;\;\;\;\;\;\;\;\;-controlrate\;at\;baseline \end{array}$$

#### Cost-effectiveness ratio

Our study applied cost-effectiveness analysis to assess the short-term economic value of MMC. In order to control confounding biases, propensity score matching (PSM) was first used to match the patients in intervention and control groups under 1:4 nearest neighbors matching, with a matching caliper of 0.05. Matching covariates included patients’ sociodemographic information (age, sex, and type of social medical insurance) and their disease conditions (diabetes type, whether or not has diabetes-related complications, duration of diabetes, whether or not use oral hypoglycemic medications/insulin, and whether or not smoke/drink alcohol). Among those covariates, social medical insurance type can be a proxy for patients’ socioeconomic status (SES). Normally, people with UE have a relatively higher SES than those with URR, as UE covers all public and private sectors’ employees and URR covers the remaining residents.

Secondly, patients’ sociodemographic information and disease conditions were reported before and after matching, and the chi-square test and Wilcoxon rank sum test were used to compare the differences between the intervention and control groups. In addition, the cost-effectiveness ratio (CER) of the two groups and the incremental cost-effectiveness ratio (ICER) were calculated based on matched data. The algorithms were:


$$\mathrm{CER}=\frac{\mathrm{total costs for diabetes management}}{\mathrm{percentage of improvement}}$$

$$\mathrm{ICER}=\frac{\mathrm{total costs for intervention group}-\mathrm{total costs for control group}}{\mathrm{improvement for intervention group}-\mathrm{improvement for control group}}$$

CER represents the unit costs for 1% control rate improvement in two groups. ICER showed the intervention group’s incremental costs for higher clinical improvement compared to the control group. The lower the CER and ICER, the higher the short-term economic value of MMC.

### Cost-utility analysis

#### Long-term costs and health outcomes

The United Kingdom Prospective Diabetes Study Outcomes Model 2 (UKPDS-OM2) was applied to simulate the long-term costs and health outcomes of the intervention and control groups. This simulation model was built on the 30 years follow-up results of the United Kingdom Prospective Diabetes Study, and was applied and validated by many previous studies (18, 19). After entering patients’ demographic, clinical risk factors and costs, as well as complication history data at present time, the model would predict individual patients’ annual incidence of death and complications (e.g., myocardial infarction, renal failure), life expectancy, QALYs and costs for diabetes management in future years (18).

Our study used patients’ clinical parameters after intervention and 1 year total costs for diabetes management to predict intervention and control groups’ QALYs and long-term costs in 50 years. The detailed data sources of clinical parameters are presented in Appendix 1 (20). In addition, the values and data sources of input therapy costs, complication costs (21–23), as well as baseline utility and utility decrements for each complication (24, 25) are shown in Appendices 2, 3. The model loop was set to be 5,000 times, and discount rates for costs and QALYs were set to be 3% as recommended by the World Health Organization (WHO) (26). Due to the requirement of UKPDS-OM2, patients with missing clinical data and/or type 1 diabetes were excluded from the simulation.

#### Cost-utility ratio

A cost-utility analysis was applied to evaluate the long-term economic value of MMC. First, PSM with nearest neighbors matching was used to match one patient from the intervention group to four patients from the control group in order to reduce confounding biases. Matching covariates included patients’ age, sex, duration of diabetes, whether or not use oral hypoglycemic medications/insulin, and whether or not smoke/drink alcohol. The matching caliper was 0.05. The differences of each covariate between intervention and control groups were tested by chi-square test or Wilcoxon rank sum test after matching. Also, our study calculated the cost-utility ratio (CUR) of each group and the incremental cost-utility ratio (ICUR) using matched data. The algorithms were:


$$\mathrm{CUR}=\frac{\mathrm{long term diabetes management costs}}{\mathrm{QALYs}}$$

$$\mathrm{ICUR}=\frac{\mathrm{long term costs for intervention group}-\mathrm{long term costs for control group}}{\mathrm{QALYs for intervention group}-\mathrm{QALYs for control group}}$$

CUR describes the unit costs per QALY for two groups, while ICUR assessed the intervention group’s incremental costs per QALY improvement compared to the control group. According to WHO’s recommendation, if ICUR was less than the annual GDP *per capita* in China (80,976 CNY in 2021), then the intervention can be considered very cost-effective. If ICUR was less than three times the annual GDP *per capita* (242,928 CNY), then the intervention is considered cost-effective. Those exceeding this level are considered not cost-effective (27, 28).

In addition, subgroup analyses for different age patients were conducted, since age can largely influence patients’ QALYs and long-term costs.

## Results

### Patients’ characteristics

A total of 6,730 patients were eligible for cost-effectiveness analysis, among which 295 patients belonged to the intervention group and 6,435 belonged to the control group. The basic characteristics differed significantly between the two groups. Compared to the control group, patients from the intervention group were younger and had shorter diabetes duration. A higher percentage of patients in the intervention group were male (intervention vs. control: 62.03% vs. 45.84%), attended UE (64.41% vs. 24.51%), had diabetes-related complications (46.78% vs. 11.97%), used insulin (24.75% vs. 16.11%), smoked (22.37% vs. 16.43%), and drank alcohol (39.32% vs. 20.14%). The detailed characteristics of patients are presented in Table 1.

**Table 1:** The basic characteristics of diabetes patients included in the study.

Variables	Intervention (*n* = 295)	Control (*n* = 6435)	*χ*^2^/*Z*	*p*
Age, years (median (iqr))^*^				
	56.00 (16.00)	67.00 (13.00)	15.86	<0.001
Duration of diabetes, months (median (iqr))^*^				
	67.00 (112.00)	104.00 (99.00)	4.25	<0.001
Sex				
Male	183 (62.03)	2950 (45.84)	29.72	<0.001
Female	112 (37.97)	3485 (54.16)		
Type of medical insurance				
URR	105 (35.59)	4858 (75.49)	231.93	<0.001
UE	190 (64.41)	1577 (24.51)		
Diabetes type				
Type 1	2 (0.68)	9 (0.14)	5.01	0.03
Type 2	293 (99.32)	6426 (99.86)		
Diabetes complication				
Don’t have	157 (53.22)	5665 (88.03)	292.91	<0.001
Have	138 (46.78)	770 (11.97)		
Oral hypoglycemic medications usage				
No	12 (4.07)	21 (0.33)	76.82	<0.001
Yes	275 (93.22)	5967 (92.73)		
Missing data	8 (2.71)	447 (6.95)		
Insulin usage				
No	214 (72.54)	5189 (80.64)	14.96	<0.001
Yes	73 (24.75)	1037 (16.11)		
Missing data	8 (2.71)	209 (3.25)		
Smoking				
No	228 (77.29)	5377 (83.56)	7.33	0.01
Yes	66 (22.37)	1057 (16.43)		
Missing data	1 (0.34)	1 (0.02)		
Drinking alcohol				
No	178 (60.34)	5138 (79.84)	63.24	<0.001
Yes	116 (39.32)	1296 (20.14)		
Missing data	1 (0.34)	1 (0.02)		

^*^Wilcoxon rank sum test. IQR-interquartile range, UE-Basic Medical Insurance for Urban Employees, URR-Basic Medical Insurance for Urban and Rural Residents.

**Figure 1:**
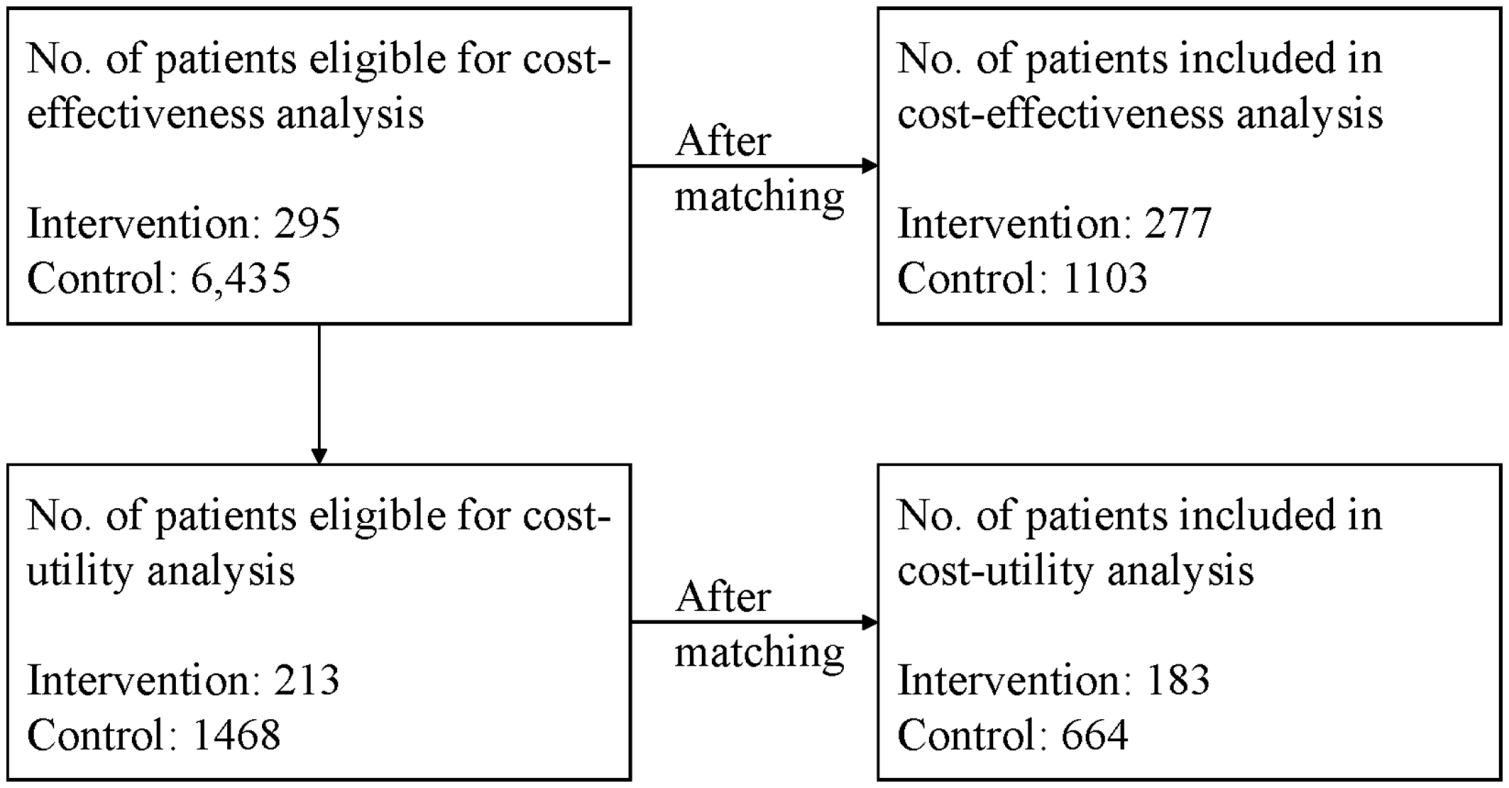
The patient flow of the study.

Among those patients, 213 patients with type 2 diabetes from the intervention group and 1,468 patients from the control group had all the clinical data required by the UKPDS-OM2 model, thus 1,681 patients were eligible for cost-utility analysis. The patient flow is shown in Figure 1.

### Cost-effectiveness analysis

#### Cost structure

After 1:4 matching, 277 patients from the intervention group and 1,103 patients from the control group were included. There were no significant differences between the two groups considering patients’ basic characteristics (Appendix 4).

The components of intervention and control groups’ 1 year diabetes management total costs are displayed in Table 2. The median (interquartile range, IQR) of total costs for the intervention group were 2536.37 (3160.18) CNY, while the total costs for control group were 2234.19 (4034.12) CNY. Among all the components, direct medical costs accounted for the largest part of total costs. For the intervention group, the direct medical costs were 1551.52 (2795.12) CNY, and for the control group, the costs were 673.08 (1378.38) CNY. In addition, the detailed components of overhead costs were shown in Appendix 5.

**Table 2:** The cost structure for 1 year diabetes management total costs.

Cost structure	Intervention	Control	Data sources
Direct medical costs (CNY)	1551.52 (2795.12)	673.08 (1378.38)	Claims dataset
Outpatient visits	6.00 (6.00)	6.00 (6.00)	
Inpatient visits	0.00 (0.00)	0.00 (0.00)	
Direct nonmedical costs per outpatient visit (CNY)	0.00 (10.00)	0.00 (10.00)	Patient questionnaires
Indirect costs per outpatient visit (CNY)	97.11 (97.11)	97.11 (97.11)	
Direct nonmedical costs per inpatient visit (CNY)	8.00 (32.00)	8.00 (32.00)	
Indirect costs per inpatient visit (CNY)	1359.48 (1747.90)	1359.48 (1747.90)	
Shared overhead costs (CNY)	–	30.65	Key informant interviews
——Intervention (Secondary hospital)	230.11	–	
——Intervention (PHC)	301.15	–	
Total costs (CNY)	2536.37 (3160.18)	2234.19 (4034.12)	

The data are presented as median (interquartile range), except for overhead costs. Total costs = direct medical costs + outpatient visits*direct nonmedical costs and indirect costs per outpatient visit + inpatient visits*direct nonmedical costs and indirect costs per inpatient visit + shared overhead costs. CNY, Chinese currency Yuan; PHC, primary healthcare center.

#### The results of cost-effectiveness analysis

The FBG control rate increased by 8.73 and 3.41% after 1 year for the intervention and control groups, respectively. Thus, for 1% FBG control rate improvement, the costs of the two groups were 290.53 CNY and 655.19 CNY. Also, the intervention group’s incremental costs per improvement (ICER) were 56.80 CNY (Table 3).

**Table 3:** The results of cost-effectiveness analysis for intervention versus control group.

Indicators	Intervention group					Control group					ICER (CNY/%)
	Costs (CNY)	Effectiveness: control rate (%)			CER (CNY/%)	Costs (CNY)	Effectiveness: control rate (%)			CER (CNY/%)	
		Baseline	After	Improvement^*^			Baseline	After	Improvement^*^		
FBG	2536.37	40.48	49.21	8.73	290.53	2234.19	45.01	48.42	3.41	655.19	56.80
SBP		50.70	71.09	20.39	124.39		80.37	84.03	3.66	610.43	18.06
DBP		73.71	83.89	10.18	249.15		91.25	92.78	1.53	1460.25	34.93

^*^Percentage of improvement = control rate after intervention-control rate at baseline. CER = total costs for diabetes management/percentage of improvement. ICER = (total costs for intervention group - total costs for control group)/(improvement for intervention group − improvement for control group). CNY, Chinese currency Yuan; CER, cost-effectiveness ratio; ICER, incremental cost-effectiveness ratio; FBG, fasting blood glucose; SBP, systolic blood pressure; DBP, diastolic blood pressure.

In addition, for 1% SBP control rate improvement, the costs of the two groups were 124.39 CNY and 610.43 CNY, leading to an ICER of 18.06 CNY/%. For 1% DBP control rate improvement, the costs of the two groups were 249.15 CNY and 1460.25 CNY, and the ICER was 34.93 CNY/%. For all three clinical indicators, the intervention group’s CERs were lower than the control group’s (Table 3).

### Cost-utility analysis

#### QALYs and long-term costs

After 1:4 matching, 183 and 664 patients from intervention and control groups were included in the cost-utility analysis. There were no significant differences between the two groups considering the basic characteristics, as shown in Appendix 6.

According to the results of the simulation model, the median (IQR) of predicted QALYs for intervention and control groups were 14.10 (4.91) and 13.80 (4.27) years, while the predicted long-term costs for the two groups were 100.00 (30.20) and 69.20 (27.40) thousand CNY. The QALYs and long-term costs decreased as patients’ age grew. In addition, in each age subgroup, the QALYs and long-term costs of the intervention group were higher than the control group (Table 4).

**Table 4:** The QALYs and long-term costs for matched intervention and control groups’ patients.

Age (year)	Intervention group		Control group	
	QALYs (years)	Long-term costs (1,000 CNY)	QALYs (years)	Long-term costs (1,000 CNY)
Total	14.10 (4.91)	100.00 (30.20)	13.80 (4.27)	69.20 (27.40)
≤49	17.74 (2.81)	117.30 (15.40)	16.89 (1.90)	81.30 (17.40)
50–59	14.76 (3.04)	99.60 (18.30)	14.09 (2.53)	66.70 (19.10)
60–69	11.96 (3.24)	80.80 (24.90)	11.61 (2.42)	51.20 (13.70)
≥70	8.27 (2.09)	59.00 (26.10)	8.12 (1.67)	44.70 (15.30)

All data are presented as median (interquartile range). QALYs, quality-adjusted life-years; CNY, Chinese currency Yuan.

#### The results of cost-utility analysis

The CURs for intervention and control groups were 7.09 and 5.01 thousand CNY/QALY, leading to an ICUR of 102.67 thousand CNY/QALY. According to WHO standards, the ICUR of the intervention group was lower than three times China’s annual GDP *per capita* (242.93 thousand CNY), thus the intervention was considered cost-effective (Table 5).

**Table 5:** The results of cost-utility analysis for intervention versus control group.

Age (year)	Intervention group			Control group			ICUR (1,000 CNY/QALY)
	Costs (1,000 CNY)	QALYs	CUR (1,000 CNY/QALY)	Costs (1,000 CNY)	QALYs	CUR (1,000 CNY/QALY)	
Total	100.00	14.10	7.09	69.20	13.80	5.01	102.67
≤49	117.30	17.74	6.61	81.30	16.89	4.81	42.35
50–59	99.60	14.76	6.75	66.70	14.09	4.73	49.10
60–69	80.80	11.96	6.76	51.20	11.61	4.41	84.57
≥70	59.00	8.27	7.13	44.70	8.12	5.50	95.33

CUR = long term diabetes management costs/QALYs. ICUR = (long term costs for intervention group-long term costs for control group)/(QALYs for Intervention group-QALYs for control group). CNY, Chinese currency Yuan; QALYs, quality-adjusted life years; CUR, cost-utility ratio; ICUR, incremental cost-utility ratio.

Subgroup analyses for different age patients were also conducted to examine the influence of age on cost-utility analysis. The results showed that for patients aged <60 years old, the ICURs for the intervention group were lower than China’s annual GDP *per capita* (80.98 thousand CNY), so the intervention was very cost-effective. But for patients aged ≥60 years old, the ICURs were lower than three times China’s annual GDP *per capita*, hence the intervention was cost-effective. Likewise, the ICUR increased as patients’ age grew (Table 5).

## Discussion

In this study, we used cost-effectiveness analysis and cost-utility analysis to evaluate the short-term and long-term economic value of MMC compared to usual diabetes management. The results showed that the intervention group’s unit costs for 1% FBG, SBP, and DBP control rate improvement were lower than the control group, which demonstrated that MMC had short-term economic value. On the other side, MMC was cost-effective considering the QALYs and long-term costs it led to, indicating that MMC had long-term economic value.

Since medical resources are always limited, health economic analysis can help policymakers to decide which treatment plan should be adopted for specific patient groups considering both the efficacy and medical costs. Our study evaluated the short-term and long-term economic value of the integrated diabetes care program, as we chose the improvement of clinical control rate and the QALYs gained after the intervention as health outcomes. In addition, the study presented a real-world situation, utilizing claim datasets and clinical information systems to collect patients’ costs and clinical improvement. Moreover, patients’ efforts and time taken to seek care were also considered in the analysis, and the costs were collected from a societal perspective.

The current study found that the intervention group’s CERs for FBG, SBP, and DBP were lower than the control group, which demonstrated that MMC’s unit costs per control rate improvement were more economic compared to usual diabetes management. This result may be explained by two reasons. First, the intervention group achieved better clinical improvement after 1 year. This may due to the fact that through standardized management plans and personalized health education, MMC motivates both medical staff and patients to participate in diabetes management actively (29). Also, the collaboration between the hospitals and the PHC, as well as the coordination between nurses and doctors provide more continuous services to patients (30). Second, the 1 year total costs of the intervention group were only a bit higher than the control group. A possible explanation for this would be that by providing high-quality outpatient services, MMC reduced patients’ hospitalization requirements and costs, thus the total costs did not increase dramatically.

Another interesting finding is that considering the long-term economic value, MMC was very cost-effective among patients aged <60 years old, and was cost-effective among patients aged ≥60 years old. It may be that these patients benefitted from better clinical management efficacy and early diabetes complication detection. Patients with diabetes complications have been found to incur twice or even triple the medical costs of those without complications (31, 32). Complications also had negative impacts on patients’ life expectancies and QALYs (33). Hence, the early detection and treatment of complications not only can reduce patients’ potential treatment costs in the future, but also improve their quality of life. Our results are also in line with those of previous studies (34), and further support the evidence that interventions involving diabetes complication screening can be cost-effective. In addition, these results also demonstrated the importance of early intervention, as MMC achieved better long-term economic value in younger patients.

Our study suggested that integrated diabetes care can be cost-effective or even very cost-effective in primary healthcare settings in China. According to the People-Centered Integrated Care (PCIC) model promoted by WHO, the directions of integrated care reform should include people empowerment and engagement, care model reorientation, and service coordination (35). Through patient education and self-management support, primary care strengthening, and the coordination between primary care and specialty care, MMC followed the principles of PCIC and improved the integration level of diabetes care. Although the intensive therapy and examinations cost additional input, MMC improved patients’ clinical conditions and QALYs, and the health economic analysis showed that the input was reasonable. Therefore, this integrated diabetes care program can be promoted in China, also its elements can be referred to by other countries to build customized integrated care models.

This study has two major limitations. First, the cost-utility analysis only included patients who had all the clinical data required by the simulation model. Those patients may have higher treatment adherence or financial resources to accept more clinical examinations compared to patients with missing data, and their clinical conditions may be better. Thus, the results of the cost-utility analysis may be overestimated. Second, our study only included patients with one-year follow-up data, introducing uncertainty into the long-term cost-utility analysis. The economic value of MMC on patients with low adherence was not examined. Thus, the results may only be extrapolated to patients with regular follow-ups. Future studies can explore the economic value of health intervention for patients with low adherence, considering the intervention’s influence on patients’ costs, efficacy, and adherence.

## Conclusion

In summary, MMC can be promoted in primary healthcare settings in China, since this integrated diabetes care program improved patients’ diabetes conditions and QALYs with reasonable costs. In addition, our study utilized real-world data to estimate the economic value of an intervention from both 1 year and lifetime horizons, which increased the extrapolation possibilities of the results. Countries that face similar diabetes management problems could also refer to the interventions implemented in the MMC program to build customized integrated care models.

## Data availability statement

The raw data supporting the conclusions of this article will be made available by the authors, without undue reservation.

## Ethics statement

The studies involving humans were approved by Medical Research Ethics Committee, School of Public Health, Fudan University. The studies were conducted in accordance with the local legislation and institutional requirements. The ethics committee/institutional review board waived the requirement of written informed consent for participation from the participants or the participants’ legal guardians/next of kin because This study used administrative data for analysis.

## Author contributions

WZ, DL, and JH designed the study. WZ conducted the analysis, interpreted the data, and wrote the first draft of the manuscript. DL revised the manuscript extensively. YD managed the implementation of the intervention program. JH and YD are the guarantor of this work and, as such, had full access to all the data in the study and takes responsibility for the integrity of the data and the accuracy of the data analysis. All authors contributed to the article and approved the submitted version.
