# Supplementary material for: A health economic analysis of an integrated diabetes care program in China: based on real-world evidence
Source: Front Public Health. 2023 Dec 19;11:1211671. doi: 10.3389/fpubh.2023.1211671 (PMC10758444; doi:10.3389/fpubh.2023.1211671)
Supplement: Supplementary file 1 [file Table_1.DOCX]

Supplementary Material

A health economic analysis of an integrated diabetes care program in China: based on real-world evidence

Wenjun Zhu, Di Liang, Jiayan Huang^*^, Yin Dong^*^

*** Correspondence:**

Jiayan Huang

Email: jiayanhuang@fudan.edu.cn

Yin Dong

Email: 9597082@qq.com

**Supplementary Table 1.** The detailed data sources of clinical parameters used in the simulation model.

| Variables | Data sources | | | |
| --- | --- | --- | --- | --- |
|  | Intervention group | Control group | | |
| Ethnicity, Sex, Age now, Duration of diabetes, Weight, Height, High-density lipoprotein, Low-density lipoprotein, Systolic blood pressure, Glycated hemoglobin, Heart rate, White blood cells, Hemoglobin | A | B | | |
| Disease history: Peripheral vascular disease, Ulcer | A | D | | |
| Disease history: Atrial fibrillation, Heart failure, Myocardial infarction, Albuminuria, Ischemic heart disease, Renal failure, Stroke; Whether or not smoke | A | B | | |
| Glomerular filtration rate^*^ | A, C (1) | B, C (1) | | |
| Disease history: Amputation, Blindness | D | | D |  |

A. MMC information system; B. county diabetes patients’ electronic health records; C. Literature; D. Assume no one happened.

^*^Glomerular filtration rate was estimated based on the patient’s serum creatinine level.

**Supplementary Table 2.** The values and data sources of input therapy costs and complication costs in the simulation model.

**Therapy costs (CNY)**

| Group | Sex | <40 Y | 40-49 Y | 50-59 Y | 60-69 Y | ≥70 Y |
| --- | --- | --- | --- | --- | --- | --- |
| Intervention | Male | 2547.15 | 1311.57 | 1609.83 | 1560.34 | 1348.69 |
|  | Female | 2257.32 | 2655.37 | 1976.39 | 1904.39 | 1365.08 |
| Control | Male | 1741.09 | 1851.07 | 1351.3 | 1177.24 | 977.49 |
|  | Female | 2432.86 | 1551.81 | 1243.63 | 1160.83 | 950.51 |

Data source: Yuhuan’s social health insurance claims dataset.

Y-years old.

**Complication costs (CNY)**

| Age (Year) | | | | | | | | | | | <40 | | | | | | | | | | | | | | | | | | | | | | [40,50) | | | | | | | | | | | | | | | | | | | [50,60) | | | | | | |  |  |  |  |  |  |  |  |  |  |
| --- | --- | --- | --- | --- | --- | --- | --- | --- | --- | --- | --- | --- | --- | --- | --- | --- | --- | --- | --- | --- | --- | --- | --- | --- | --- | --- | --- | --- | --- | --- | --- | --- | --- | --- | --- | --- | --- | --- | --- | --- | --- | --- | --- | --- | --- | --- | --- | --- | --- | --- | --- | --- | --- | --- | --- | --- | --- | --- | --- | --- | --- | --- | --- | --- | --- | --- | --- | --- |
|  |  |  |  |  |  |  |  |  |  |  | **At time of event** | | | | | | | | | | **Cost in subsequent years** | | | | | | | **At time of event** | | | | | | | | | | | | | | | **Cost in subsequent years** | | | | | | | | | | **At time of event** | | | | | | | | | | | | | | | **Cost in subsequent years** |
| **Male** | **Fatal cost** | | | | | **Non-fatal cost** | | | | | **Fatal cost** | | | | | | | **Non-fatal cost** | | | | | | | |  |  | **Fatal cost** | | | | | | | | | **Non-fatal cost** | | | | | |  |  |  |  |  |  |  |  |  |  |  |  |  |  |  |  |  |  |  |  |  |  |  |  |  |  |
| **Ischemic heart disease (2)** | 3810.00 | | | | | 10557.00 | | | | | 9048.00 | | | | | | | | | | 3690.00 | | | | | | | | | | | | 10224.00 | | | | | | | | | | 8764.00 | | | | 4096.00 | | | | | 11350.00 | | | | | | 9732.00 |  |  |  |  |  |  |  |  |  |  |
| **Myocardial infarction (2)** | | | | | | 20446.00 | | | | | | | 30355.00 | | | | | | 9236.00 | | | | | | | | | 19803.00 | | | | | | | 29400.00 | | | | | | 8944.00 | | | | | | | | | 21982.00 | | | | | 32638.00 | | | 9928.00 |  |  |  |  |  |  |  |  |  |  |
| **Heart failure (2)** | | | | | | 6106.00 | | | | | 14860.00 | | | | | 10812.00 | | | | | | | | 5914.00 | | | | | | 14392.00 | | | | | | | | 10472.00 | | | | | | | | 6565.00 | | | | | | 15976.00 | | | | 11624.00 | | |  |  |  |  |  |  |  |  |  |  |
| **Stroke (2)** | 11449.00 | | 22795.00 | | | | | | | 12784.00 | | | | | 11088.00 | | | | | | | 22077.00 | | | | | | | | | | 12384.00 | | | | | | | 12309.00 | | | | | | | | | 24507.00 | | | | | | | | | | 13748.00 |  |  |  |  |  |  |  |  |  |  |
| **Amputation (3)** | 45951.78 | | | | | | 45951.78 | | | | | 9996.00 | | | | | 45951.78 | | | | | | | | | | 45951.78 | | | | | | | 9996.00 | | | | | | | | 45951.78 | | | | | | | | | | | 45951.78 | | | | | 9996.00 |  |  |  |  |  |  |  |  |  |  |
| **Blindness (4)** | | 0.00 | | | 4438.82 | | | | | | | | | 1202.95 | | | | | | 0.00 | | | | | | 4438.82 | | | | | | | | | | | 1202.95 | | | | | | | | 0.00 | | | | 4438.82 | | | | | | | | 1202.95 | |  |  |  |  |  |  |  |  |  |  |
| **Renal failure (2)** | | 3460.00 | | | | | | 12073.00 | | | | | | 11596.00 | | | | | | 3351.00 | | | | | | | | | 11691.00 | | | | | | | | 11228.00 | | | | | | | | 3720.00 | | | | | | 12978.00 | | | | | | 12464.00 | |  |  |  |  |  |  |  |  |  |  |
| **Ulcer (2)** | 2915.00 | | | 10784.00 | | | | | 9996.00 | | | | | | 2823.00 | | | | | | | | 10443.00 | | | | | | | | | | | | | 9680.00 | | | | | | | | 3134.00 | | | | | | | | | | 11594.00 | | | | 10748.00 |  |  |  |  |  |  |  |  |  |  |
| **Female** | | | |  | | | | |  | | | | | |  | | | | | | | |  | | | | | | | |  | | | | | | | | |  | | | | | | |  | | | | | | |  | | | |  |  |  |  |  |  |  |  |  |  |  |
| **Ischemic heart disease** | | | | 3674.00 | | | | | 10181.00 | | | | | | 8728.00 | | | | | | | | 3559.00 | | | | | | | | 9862.00 | | | | | | | | | 8456.00 | | | | | | | 3950.00 | | | | | | | 10946.00 | | | | 9384.00 |  |  |  |  |  |  |  |  |  |  |
| **Myocardial infarction** | | | | 19720.00 | | | | | 29278.00 | | | | | | 8908.00 | | | | | | | | 19100.00 | | | | | | | | 28358.00 | | | | | | | | | 8628.00 | | | | | | | 21202.00 | | | | | | | 31480.00 | | | | 9576.00 |  |  |  |  |  |  |  |  |  |  |
| **Heart failure** | | | | 5889.00 | | | | | 14331.00 | | | | | | 10428.00 | | | | | | | | 5704.00 | | | | | | | | 13882.00 | | | | | | | | | 10100.00 | | | | | | | 6332.00 | | | | | | | 15410.00 | | | | 11212.00 |  |  |  |  |  |  |  |  |  |  |
| **Stroke** | | | | 11042.00 | | | | | 21986.00 | | | | | | 12332.00 | | | | | | | | 10695.00 | | | | | | | | 21294.00 | | | | | | | | | 11944.00 | | | | | | | 11872.00 | | | | | | | 23638.00 | | | | 13256.00 |  |  |  |  |  |  |  |  |  |  |
| **Amputation** | | | | 45951.78 | | | | | 45951.78 | | | | | | 9996.00 | | | | | | | | 45951.78 | | | | | | | | 45951.78 | | | | | | | | | 9996.00 | | | | | | | 45951.78 | | | | | | | 45951.78 | | | | 9996.00 |  |  |  |  |  |  |  |  |  |  |
| **Blindness** | | | | 0.00 | | | | | 4438.82 | | | | | | 1202.95 | | | | | | | | 0.00 | | | | | | | | 4438.82 | | | | | | | | | 1202.95 | | | | | | | 0.00 | | | | | | | 4438.82 | | | | 1202.95 |  |  |  |  |  |  |  |  |  |  |
| **Renal failure** | | | | 3337.00 | | | | | 11644.00 | | | | | | 11184.00 | | | | | | | | 3232.00 | | | | | | | | 11278.00 | | | | | | | | | 10832.00 | | | | | | | 3588.00 | | | | | | | 12519.00 | | | | 12024.00 |  |  |  |  |  |  |  |  |  |  |
| **Ulcer** | | | | 2811.00 | | | | | 10401.00 | | | | | | 9640.00 | | | | | | | | 2723.00 | | | | | | | | 10073.00 | | | | | | | | | 9336.00 | | | | | | | 3022.00 | | | | | | | 11182.00 | | | | 10364.00 |  |  |  |  |  |  |  |  |  |  |

| Age (Year) | [60,70) | | | [70,80) | | | ≥80 | | |
| --- | --- | --- | --- | --- | --- | --- | --- | --- | --- |
|  | **At time of event** | | **Cost in subsequent years** | **At time of event** | | **Cost in subsequent years** | **At time of event** | | **Cost in subsequent years** |
| **Male** | **Fatal cost** | **Non-fatal cost** |  | **Fatal cost** | **Non-fatal cost** |  | **Fatal cost** | **Non-fatal cost** |  |
| **Ischemic heart disease** | 4341.00 | 12030.00 | 10312.00 | 4374.00 | 12120.00 | 10392.00 | 4333.00 | 12007.00 | 10292.00 |
| **Myocardial infarction** | 23299.00 | 34591.00 | 10524.00 | 23478.00 | 34857.00 | 10604.00 | 23254.00 | 34525.00 | 10504.00 |
| **Heart failure** | 6958.00 | 16933.00 | 12324.00 | 7011.00 | 17064.00 | 12416.00 | 6945.00 | 16902.00 | 12300.00 |
| **Stroke** | 13046.00 | 25976.00 | 14568.00 | 13146.00 | 26175.00 | 14680.00 | 13021.00 | 25924.00 | 14540.00 |
| **Amputation** | 45951.78 | 45951.78 | 9996.00 | 45951.78 | 45951.78 | 9996.00 | 45951.78 | 45951.78 | 9996.00 |
| **Blindness** | 0.00 | 4438.82 | 1202.95 | 0.00 | 4438.82 | 1202.95 | 0.00 | 4438.82 | 1202.95 |
| **Renal failure** | 3942.00 | 13755.00 | 13212.00 | 3973.00 | 13861.00 | 13316.00 | 3935.00 | 13730.00 | 13188.00 |
| **Ulcer** | 3321.00 | 12288.00 | 11392.00 | 3347.00 | 12383.00 | 11476.00 | 3315.00 | 12264.00 | 11368.00 |
| **Female** |  |  |  |  |  |  |  |  |  |
| **Ischemic heart disease** | 4187.00 | 11603.00 | 9948.00 | 4219.00 | 11692.00 | 10024.00 | 4179.00 | 11580.00 | 9928.00 |
| **Myocardial infarction** | 22472.00 | 33365.00 | 10152.00 | 22644.00 | 33621.00 | 10228.00 | 22428.00 | 33300.00 | 10132.00 |
| **Heart failure** | 6711.00 | 16332.00 | 11884.00 | 6762.00 | 16458.00 | 11976.00 | 6698.00 | 16301.00 | 11860.00 |
| **Stroke** | 12583.00 | 25054.00 | 14052.00 | 12680.00 | 25244.00 | 14160.00 | 12559.00 | 25006.00 | 14024.00 |
| **Amputation** | 45951.78 | 45951.78 | 9996.00 | 45951.78 | 45951.78 | 9996.00 | 45951.78 | 45951.78 | 9996.00 |
| **Blindness** | 0.00 | 4438.82 | 1202.95 | 0.00 | 4438.82 | 1202.95 | 0.00 | 4438.82 | 1202.95 |
| **Renal failure** | 3802.00 | 13267.00 | 12744.00 | 3832.00 | 13369.00 | 12840.00 | 3795.00 | 13242.00 | 12720.00 |
| **Ulcer** | 3203.00 | 11852.00 | 10984.00 | 3228.00 | 11943.00 | 11072.00 | 3197.00 | 11828.00 | 10964.00 |

Data source: Literature.

**Supplementary Table 3**. The values and data sources of baseline utility and utility decrements for each complication in the simulation model.

| Disease | Utility |
| --- | --- |
| Initial utility (without complications) | 0.920 (5) |
| Ischemic heart disease | -0.017 (5) |
| Myocardial infarction | -0.007 (5) |
| Heart failure | -0.050 (5) |
| Stroke | -0.142 (6) |
| Amputation | -0.177 (5) |
| Blindness | -0.101 (5) |
| Renal failure | -0.055 (5) |
| Ulcer | -0.094 (5) |

**Supplementary Table 4.** The basic characteristics of matched patients included in cost-effectiveness analysis.

| Variables | Intervention (n=277) | Control (n=1103) | χ^2^/Z | *P* |
| --- | --- | --- | --- | --- |
| Age, years (median (IQR)) ^*^ | |  |  |  |
|  | 56.00 (14.00) | 56.00 (13.00) | 0.57 | 0.57 |
| Duration of diabetes, months (median (IQR)) ^*^ | | |  |  |
|  | 67.00 (113.00) | 76.00 (94.00) | 0.20 | 0.85 |
| Sex |  |  |  |  |
| Male | 174 (62.82) | 687 (62.28) | 1.28 | 0.53 |
| Female | 103 (37.18) | 416 (37.72) |  |  |
| Type of medical insurance | |  |  |  |
| URR | 101 (36.46) | 421 (38.17) | 1.53 | 0.47 |
| UE | 176 (63.54) | 682 (61.83) |  |  |
| Diabetes type |  |  |  |  |
| Type 1 | 1 (0.36) | 3 (0.27) | 1.32 | 0.52 |
| Type 2 | 276 (99.64) | 1100 (99.73) |  |  |
| Diabetes complication | |  |  |  |
| Don't have | 150 (54.15) | 599 (54.31) | 1.26 | 0.53 |
| Have | 127 (45.85) | 504 (45.69) |  |  |
| Oral hypoglycemic medications usage | | |  |  |
| No | 6 (2.17) | 14 (1.27) | 2.50 | 0.29 |
| Yes | 271 (97.83) | 1089 (98.73) |  |  |
| Missing data | 0 (0.00) | 0 (0.00) |  |  |
| Insulin usage |  |  |  |  |
| No | 212 (76.53) | 876 (79.42) | 2.36 | 0.31 |
| Yes | 65 (23.47) | 227 (20.58) |  |  |
| Missing data | 0 (0.00) | 0 (0.00) |  |  |
| Smoking |  |  |  |  |
| No | 212 (76.53) | 846 (76.70) | 1.26 | 0.53 |
| Yes | 65 (23.47) | 257 (23.30) |  |  |
| Missing data | 0 (0.00) | 0 (0.00) |  |  |
| Drinking alcohol |  |  |  |  |
| No | 168 (60.65) | 667 (60.47) | 1.26 | 0.53 |
| Yes | 109 (39.35) | 436 (39.53) |  |  |
| Missing data | 0 (0.00) | 0 (0.00) |  |  |

^*^Wilcoxon rank sum test

IQR-interquartile range, UE-Basic Medical Insurance for Urban Employees, URR-Basic Medical Insurance for Urban and Rural Residents.

**Supplementary Table 5**. The detailed components of overhead costs.

| Group | Items | Costs (CNY) | Shared unit |
| --- | --- | --- | --- |
| Intervention (secondary hospital) | Clinic re-decoration | 539191.00 | Depreciation by 15 years；the number of MMC enrollees |
|  | Diabetes nurse income | 190000.00 | The number of MMC enrollees |
|  | AI follow-up | 25000.00 |  |
|  | Health education | 69600.00 |  |
|  | Receiving training | 4738.46 |  |
|  | Providing training | 30461.54 |  |
| Intervention (PHC) | Clinic re-decoration | 95000.00 | Depreciation by 15 years；the number of MMC enrollees |
|  | Diabetes nurse income | 105000.00 | The number of MMC enrollees |
|  | Health education | 81400.00 |  |
| Control | Health education | 42980.00 | The number of residents |
|  | Receiving training | 35496.98 |  |
|  | fee-for-free follow-up per time | 30.25 |  |

CNY-Chinese Currency Yuan; PHC-primary healthcare center.

**Supplementary Table 6**. The basic characteristics of matched patients included in cost-utility analysis.

| Variables | Intervention (n=183) | Control (n=664) | χ^2^/Z | *P* |
| --- | --- | --- | --- | --- |
| Age, years (median (IQR)) ^*^ | |  |  |  |
|  | 55.00 (11.00) | 56.00 (11.00) | 0.19 | 0.85 |
| Duration of diabetes, months (median (IQR)) ^*^ | | |  |  |
|  | 6.58 (9.75) | 7.17 (6.92) | 0.31 | 0.76 |
| Sex |  |  |  |  |
| Male | 141 (77.05) | 495 (74.55) | 0.48 | 0.49 |
| Female | 42 (22.95) | 169 (25.45) |  |  |
| Oral hypoglycemic medications usage | | |  |  |
| No | 5 (2.73) | 7 (1.05) | 2.89 | 0.09 |
| Yes | 178 (97.27) | 657 (98.95) |  |  |
| Insulin usage |  |  |  |  |
| No | 139 (75.96) | 538 (81.02) | 2.30 | 0.13 |
| Yes | 44 (24.04) | 126 (18.98) |  |  |
| Smoking |  |  |  |  |
| No | 124 (67.76) | 437 (65.81) | 0.24 | 0.62 |
| Yes | 59 (32.24) | 227 (34.19) |  |  |
| Drinking alcohol |  |  |  |  |
| No | 87 (47.54) | 328 (49.40) | 0.20 | 0.66 |
| Yes | 96 (52.46) | 336 (50.60) |  |  |

^*^Wilcoxon rank sum test

IQR-interquartile range.

**References**

1. Shi H, Chen N, Zhang W, Ren H, Xu Y, Shen P, et al. Evaluating and Refitting the Simplified Equation of Mdrd to Predict Glomerular Filtration Rate in Chinese Patients with Chronic Kidney Disease (in Chinese). *Chinese Journal of Practical Internal Medicine* (2006) (09):665-9.

2. He X, Zhang Y, Zhou Y, Dong C, Wu J. Direct Medical Costs of Incident Complications in Patients Newly Diagnosed with Type 2 Diabetes in China. *Diabetes Ther* (2021) 12(1):275-88. Epub 20201118. doi: 10.1007/s13300-020-00967-y.

3. Lu Q, Wang J, Wei X, Wang G, Xu Y, Lu Z, et al. Cost of Diabetic Foot Ulcer Management in China: A 7-Year Single-Center Retrospective Review. *Diabetes Metab Syndr Obes* (2020) 13:4249-60. Epub 20201110. doi: 10.2147/dmso.S275814.

4. Wu J, Zheng Y. Cost-Effectiveness Analysis of Biphasic Insulin Aspart 30 and Premixed Human Insulin in Chinese Patients with Type 2 Diabetes (in Chinese). *Chinese Pharmaceutical Journal* (2010) 45(14):1116-20.

5. Mok CH, Kwok HHY, Ng CS, Leung GM, Quan J. Health State Utility Values for Type 2 Diabetes and Related Complications in East and Southeast Asia: A Systematic Review and Meta-Analysis. *Value Health* (2021) 24(7):1059-67. Epub 20210410. doi: 10.1016/j.jval.2020.12.019.

6. Shi Z, Dong Y, Li S. Health State Utility Values for Diabetes Patients in China: A Systematic Review and Meta-Analysis (in Chinese). *Modern Preventive Medicine* (2022) 49(06):1091-8.
